# Efficacy and Limitations of Corneal Confocal Microscopy in Evaluating ATTRv‐PN

**DOI:** 10.1002/brb3.71705

**Published:** 2026-08-25

**Authors:** Yize Li, Kang Du, Wenjing Song, Difang Shi, Xujun Chu, Ping Li, Zhenyu Li, He Lv, Wei Zhang, Zhaoxia Wang, Yun Yuan, Yuan Wu, Lingchao Meng

**Affiliations:** ^1^ Department of Neurology Peking University First Hospital Beijing China; ^2^ Department of Neurology Affiliated Qujing Hospital of Kunming Medical University/Qujing Central Hospital of Yunnan Province Qujing Yunnan China; ^3^ Department of Ophthalmology Peking University First Hospital Beijing China; ^4^ Department of Thoracic Surgery I The Third Affiliated Hospital of Kunming Medical University (Yunnan Cancer Hospital) Kunming Yunnan China; ^5^ Department of Neurology The Affiliated Shandong Provincial Hospital of Shandong First Medical University Jinan Shandong China; ^6^ Children Vision Institute Peking University Beijing China

**Keywords:** ATTRv‐PN, corneal confocal microscopy, hereditary transthyretin amyloidosis

## Abstract

**Objective:**

Driven by pathogenic variants in the *TTR* gene, hereditary transthyretin amyloidosis with polyneuropathy (ATTRv‐PN) presents as a systemic autosomal dominant disorder. This cross‐sectional study aimed to investigate the clinical utility of in vivo corneal confocal microscopy (CCM) for assessing structural changes in corneal nerve fibers among ATTRv‐PN patients and presymptomatic carriers.

**Methods:**

A total of 41 ATTRv‐PN patients, 15 ATTRv‐PN presymptomatic carriers, and 15 healthy controls underwent CCM assessment. Clinical neurological scores, electrophysiological data, and sural biopsy data from ATTRv‐PN patients and carriers were analyzed. We calculated the corneal nerve fiber length (CNFL), corneal nerve fiber density (CNFD), corneal nerve branch density (CNBD), inferior whorl fiber length (IWFL), inferior whorl fiber density (IWFD), inferior whorl branch density (IWBD), corneal Langerhans cell density (CLCD), and inferior whorl Langerhans cell density (IWLCD). We compared the CCM parameters between patients, carriers, and healthy controls and analyzed their correlations with clinical, electrophysiological, and pathological changes in patients with ATTRv‐PN.

**Results:**

CCM parameters, including CNFL, CNFD, CNBD, IWFL, CLCD, and IWLCD, were abnormal in ATTRv‐PN patients (*p* < 0.05). Statistical analysis revealed no significant variation in evaluated CCM parameters between the early‐onset and late‐onset ATTRv‐PN patient subgroups. Similarly, there were not statistically significant differences in these parameters between Stage I patients and Stage II patients. All CCM parameters except CNFD and IWFL were abnormal in presymptomatic carriers (*p <* 0.05). The CNFL and IWLCD (*p <* 0.05) were correlated with disease duration. The CNFL, CNFD, and CNBD were positively correlated with sural large myelinated nerve fiber density (*p* < 0.05), so were CNFL and IWFL with the total myelinated nerve fiber density (*p* < 0.05) in the sural nerve.

**Conclusion:**

As an in vivo, noninvasive imaging technique, CCM facilitates the detailed evaluation of corneal nerve fiber morphology in both symptomatic ATTRv‐PN individuals and asymptomatic mutation carriers. We have confirmed the significant abnormalities of CCM parameters of corneal nerve fibers in ATTRv‐PN patients and presymptomatic carriers.

## Graphical abstract

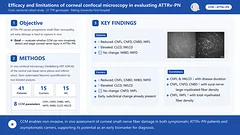

## 1 Introduction

Triggered by pathogenic mutations in the *TTR* gene, hereditary transthyretin amyloidosis with polyneuropathy (ATTRv‐PN) presents as a rare, autosomal dominant genetic disorder (Schmidt et al. 2018; Thimm et al. 2023; Jiao et al. 2025), which is characterized by the involvement of peripheral nervous system, central nervous system, heart, eyes, skin, muscle, and kidneys (Adams, Ando et al. 2021). The phenotypic features of ATTRv‐PN vary largely from case to case (Jiao et al. 2025; Minnella et al. 2021). The disease is stratified by the age of initial symptom presentation into early‐onset (occurring before age 50) and late‐onset (occurring at or after age 50) type. The average survival time after symptom onset ranges from 2 to 15 years (Zhang et al. 2021; Coelho et al. 2018). So far, over 130 pathogenic *TTR* variants have been documented globally, with distinct geographical distributions. Recently, innovative pharmacological interventions, such as antisense oligonucleotide (ASO) and small interfering RNA (siRNA) drugs, have proved to be effective in the early stages (Zhang et al. 2021; Adams, Polydefkis et al. 2021; Plante‐Bordeneuve 2018). Identifying reliable early biomarkers for the diagnosis of ATTRv‐PN patients and presymptomatic carriers is of great clinical significance.

The corneal confocal microscopy (CCM) is a rapid, noninvasive technique to detect corneal nerve morphology and has been used in a range of neurological diseases (Bitirgen et al. 2018; Al‐Janahi et al. 2020; Dhage et al. 2021; Petropoulos et al. 2021; Toprak et al. 2023). Given that corneal nerve fibers consist of small fibers, CCM provides a reliable approach to the assessment of small fiber neuropathies. Earlier studies have demonstrated the reduction of corneal nerve fibers associated with diabetic peripheral neuropathy (Xiong et al. 2018). In patients with AL amyloidosis, a marked loss of corneal small nerve fibers has been observed, even in the absence of large fiber involvement (Thimm et al. 2022). It has been found that ATTRv‐PN starts with an impact on small‐fiber nerves (Carroll et al. 2022; Planté‐Bordeneuve and Said 2011). Three previous studies have employed CCM to evaluate small‐fiber nerves in ATTRv‐PN (Thimm et al. 2023; Zhang et al. 2021; Alcantara et al. 2024). However, these studies were beset with limitations. Specifically, the sample sizes were relatively small, the genotypes were rather homogeneous, and no correlation analysis was conducted in relation to neuropathology, which was why this study was intended to find out about the characteristics of CCM in patients with ATTRv‐PN and carriers. Additionally, it endeavored to explore the associations between CCM and clinical as well as neuropathological indicators.

## 2 Methods

### 2.1 Subjects

This study included a total of 41 ATTRv‐PN patients diagnosed between 2018 and 2024, 15 presymptomatic carriers, and 15 healthy controls (HC). Healthy controls were prospectively selected to match the age and sex distribution of the ATTRv‐PN patients and presymptomatic carriers. The patient cohort was stratified into an early‐onset subgroup (onset age < 50 years) and a late‐onset subgroup (onset age ≥ 50 years). Patients were divided into the clinical Stage I and Stage II group according to Coutinho stage. Those in the healthy control group had no documented history of diabetes, neurological deficits, or diseases predisposing them to peripheral neuropathy. Additionally, ocular exclusion criteria included active or historical corneal diseases, previous eye surgeries or trauma, and prior wear of contact lenses.

All participants received thorough clinical assessments. Neurological examinations involved comprehensive sensory‐motor function tests and reflex testing of all limbs, with lower limb impairment specifically scored via the Neuropathy Impairment Score in the Lower Limbs (NIS‐LL). The Composite Autonomic Symptom Score with a remaining total of 31 questions in 6 domains (COMPASS‐31) index was adopted to assess autonomic nerve system involvement. Moreover, electrophysiological nerve conduction studies were executed for all symptomatic individuals and presymptomatic carriers.

Relevant therapeutic data were extracted for all patients. Patients were subsequently stratified into treated and treatment‐naïve subgroups for further analysis.

### 2.2 Corneal Confocal Microscopy Study

All corneal imaging was acquired via an in vivo confocal microscopic system (Heidelberg Retina Tomograph II/Rostock Corneal Module; Heidelberg Engineering, Heidelberg, Germany) by the same examiner by focusing scanning on the central sub‐basal corneal nerve plexus and the characteristic inferior whorl‐like nerve plexus. For image selection, four high‐quality conventional nerve images and three clear images of the inferior whorl region were selected from each eye, with a total of seven images analyzed per eye. Only images with accurate focusing, favorable contrast, intact nerve structure and no motion artifacts, epithelial lesions or light reflection interference were included, while blurred or artifact‐contaminated images were excluded and re‐acquired. All CCM images were captured by a single experienced technician. Two independent image analysts were fully blinded to participants’ grouping, clinical diagnosis and disease stage during quantification, and all original images were retained for subsequent review and quality control. All corneal nerve parameters, including corneal nerve fiber length (CNFL), corneal nerve fiber density (CNFD), corneal nerve branch density (CNBD), inferior whorl fiber length (IWFL), inferior whorl fiber density (IWFD), inferior whorl branch density (IWBD), corneal Langerhans cell density (CLCD), and inferior whorl Langerhans cell density (IWLCD) were semi‐automatically quantified using ImageJ software with the Neuron J plugin. As the unique whorled arrangement of nerve fibers in the inferior whorl region makes reliable assessment impossible for fully automated analysis, we adopted a semi‐automatic protocol where analysts manually traced the main nerve fibers and branches, and the software automatically calculated the number of nerve fibers, branch counts and total nerve length per unit area. Additionally, we assessed intra‐rater and inter‐rater reproducibility by randomly reanalyzing 20% of all the samples. Intra‐rater reliability was evaluated via repeated measurements by the same analyst at a 14‐day interval while inter‐rater reliability was determined by comparing results from the two independent analysts using the coefficient of variation (CV). All intra‐ and inter‐rater CV values were below 10%, with lower CVs observed for CNFL, CNFD, IWFL compared with other indices, confirming excellent stability and reproducibility of our measurements. The measurement and calculation of the CCM parameters were conducted in accordance with the methodologies described in previous studies (Thimm et al. 2023; Zhang et al. 2021; Rousseau et al. 2016).

### 2.3 Sural Biopsy

Following toluidine blue staining, semithin sections were evaluated via light microscopy to determine morphometric parameters. Given the variability among fascicles, myelinated fibers were manually quantified across a minimum of three distinct fascicles using complete transverse sural nerve micrographs obtained at 400× magnification to encompass the broadest possible nerve cross‐section. Subsequently, the respective myelinated nerve fiber densities for the overall, large (> 7 µm), and small (≤ 7 µm) nerve fibers were computed with Adobe Photoshop CC 2018 software (San Jose, CA).

### 2.4 Statistical Analysis

All statistical analysis was conducted using GraphPad Prism. The Anderson–Darling method was employed to evaluate data normality. CCM parameters, demographic data, neuropathology data and clinical data were all subject to non‐normal distributions. All data were analyzed using nonparametric tests. The Mann–Whitney test was conducted to compare distributions between two groups of data. Significant distributional differences across multiple groups were studied via the Kruskal–Wallis test, followed by the Dunn test to appropriately correct for multiple testing. The Spearman rank‐order correlation coefficient was computed to identify the correlations between two variables. For subgroup and correlation analyses, strict multiple testing corrections were not applied across all parameters to avoid an inflated Type II error rate due to the design of this study and the restricted sample size of rare disease study. Instead, exact *p* values were reported, and findings with borderline significance (0.01 < *p* < 0.05) were interpreted with caution.

## 3 Results

### 3.1 Clinical Data

Demographic data of the 41 patients, 15 carriers, and 15 healthy controls are given in Table 1. The male‐to‐female ratio was 31:10 for ATTRv‐PN, 11:4 for carriers, and 9:6 for HC. No statistically significant differences were detected between the three groups in age or sex distribution (Table 1). The average disease duration of ATTRv‐PN patients was 3.96 ± 3.22 years (Table 2). For the Coutinho stages, 24 (58.5%) patients were in Stage I, while 17 (41.5%) were in Stage II. The average scores of NIS‐LL and COMPASS‐31 were 58.86 ± 37.00 and 39.93 ± 22.86, respectively. Among patients, 11 had the V30M mutation type, and 30 had the non‐V30M mutation type. Among carriers, four had the V30M mutation type, and 11 had the non‐V30M mutation type (**Table** **3**).

**TABLE 1:** Demography of patients with ATTRv‐PN, presymptomatic carriers and healthy controls (HC).

Parameter	Patients *n* = 41	Carriers *n* = 15	HC *n* = 15	*p* value ATTRv‐PN vs. HC	*p* value Carriers vs. HC	*p* value ATTRv‐PN vs. carriers
Gender (male: female)	31:10	11:4	9:6	0.311	0.682	1.000
Age, years	53.22 ± 13.03	40.07 ± 10.74	45.4 ± 9.60	0.920	0.823	0.057

**TABLE 2:** Clinical characteristics of patients with ATTRv‐PN.

Clinical characteristics	Patients *n* = 41
Age of disease onset, years	49.24 ± 12.89
Early onset: late onset, count (percentage)	19(46.3%):22(53.7%)
Disease duration, years	3.96 ± 3.22
Coutinho stages (ATTRv‐PN), count (percentage)	Stage I: 24 (58.5%); Stage II: 17 (41.5%)
NIS‐LL	58.86 ± 37.00
COMPASS‐31	39.93 ± 22.86

Abbreviations: COMPASS‐31, Composite Autonomic Symptom Score with a remaining total of 31 questions in 6 domains; NIS‐LL, Neuropathy Impairment Score in the Lower Limbs.

**TABLE 3:** *TTR* gene mutation types in patients and carriers.

Mutations	Patients	Carriers	Sum
	*n* = 41	*n* = 15	*n* = 56
V30M	11	4	15
V30L	4	3	7
A97S	3	2	5
G83R	1	3	4
S77F	2	1	3
E61K	3	0	3
K35N	3	0	3
E42G	2	0	2
G47R	2	0	2
F33L	0	1	1
T49A	0	1	1
A36P	1	0	1
D38V	1	0	1
E54G	1	0	1
F33V	1	0	1
L55P	1	0	1
S77Y	1	0	1
T59K	1	0	1
V28S	1	0	1
V30A	1	0	1
Y114C	1	0	1

By the time of CCM evaluation, 23 patients had received disease‐modifying treatments (12 on diflunisal, 10 on tafamidis, and 1 had undergone liver transplantation), while the remaining 18 patients were treatment‐naïve.

### 3.2 CCM Parameters in Patients

**FIGURE 1:**
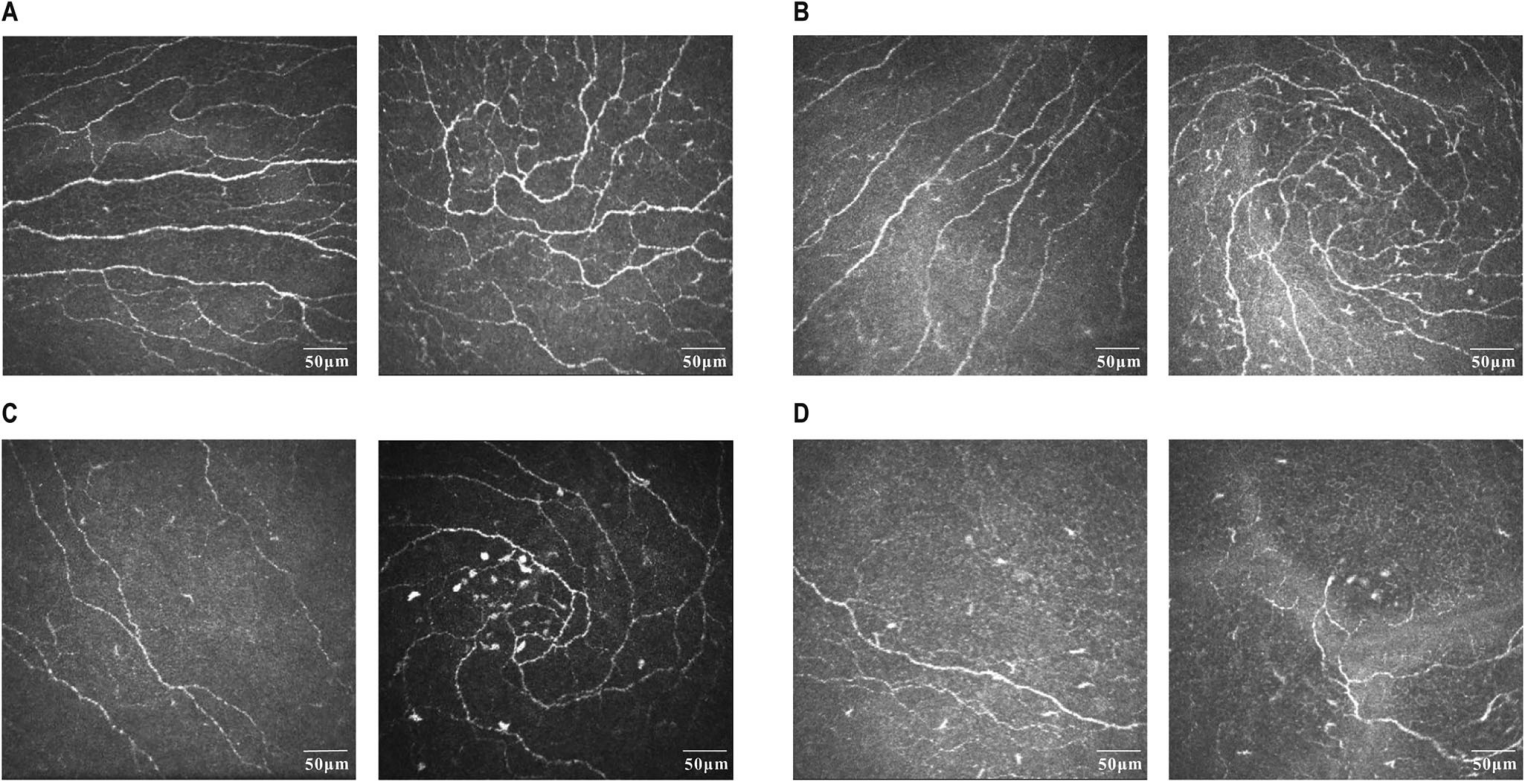
Corneal confocal microscopy images of the cornea and inferior whorl in HC (A), presymptomatic ATTRv‐PN carriers (B), Stage I patients (C), and Stage II patients (D). Corneal nerve fiber loss in ATTRv‐PN patients was clearly observed. Clustering of Langerhans cells is observable in both preclinical carriers and patient groups (particularly prominent presentation in Stage I patients).

The CCM data are presented in Table 4. All CCM parameters except IWBD and IWFD in ATTRv‐PN patients were significantly abnormal (Figure 1). The CNFL (*p* < 0.0001), CNFD (*p* = 0.0004), CNBD (*p* = 0.0002), and IWFL (*p* = 0.0007) showed a substantial decrease in patients compared with HC. Furthermore, IWFD (*p* = 0.19) and IWBD (*p* = 0.11) showed no difference in patients compared to the HC group. The CLCD (*p* = 0.03) and IWLCD (*p* = 0.01) were notably elevated in patients compared to HC.

**TABLE 4:** Parameters of corneal confocal microscopy in ATTRv‐PN, carriers and HC (mean ± SD).

Parameters	ATTRv‐PN *n* = 41	Carriers *n* = 15	HC *n* = 15	*p* value ATTRv‐PN vs. HC	*p* value Carriers vs. HC	*p* value ATTRv‐PN vs. carriers
CNFL (mm/mm^2^)	9.70 ± 5.37	15.14 ± 3.35	18.13 ± 3.10	< 0.0001****	0.02*	0.0005***
CNFD (no./mm^2^)	23.60 ± 10.37	31.22 ± 7.05	33.96 ± 6.43	0.0004***	0.26	0.007**
CNBD (no./mm^2^)	25.70 ± 21.02	30.00 ± 12.40	47.08 ± 13.86	0.0002***	0.002**	0.15
IWFL (mm/mm^2^)	10.41 ± 5.67	13.75 ± 5.23	16.37 ± 2.31	0.0007***	0.09	0.08
IWFD (no./mm^2^)	21.88 ± 8.53	20.51 ± 7.39	24.58 ± 3.71	0.19	0.04*	0.64
IWBD (no./mm^2^)	38.40 ± 33.16	36.82 ± 18.67	47.50 ± 12.39	0.11	0.02*	0.58
CLCD (no./mm^2^)	29.15 ± 27.47	44.38 ± 29.32	14.27 ± 10.18	0.03*	< 0.0001****	0.02*
IWLCD (no./mm^2^)	48.28 ± 58.63	56.45 ± 67.07	16.88 ± 14.99	0.01*	0.0007***	0.23

Abbreviations: CLCD, corneal Langerhans cell density; CNBD, corneal nerve branch density; CNFD, corneal nerve fiber density; CNFL, corneal nerve fiber length; IWBD, inferior whorl nerve branch density; IWFD, inferior whorl fiber density; IWFL; inferior whorl corneal nerve fiber length. IWLCD; inferior whorl Langerhans cell density.

* *p* < 0.05.

^**^ *p* < 0.01.

^***^ *p* < 0.001.

^****^ p < 0.0001.

Statistical analyses revealed no statistically significant differences (*p* > 0.05) in CCM parameters (CNFL, CNFD, CNBD, IWFL, IWFD, IWBD, CLCD, and IWLCD) between clinical Stage I and Stage II patients. In the genetic mutation subgroup analysis, patients with the V30M mutation had marginally lower CLCD levels than the non‐V30M mutation group (*p* = 0.049). Similarly, statistical analyses did not find significant differences (all *p* > 0.05) in CCM parameters between early‐onset and late‐onset ATTRv‐PN patients.

To assess the potential impact of therapies, a subgroup analysis was performed to compare CCM parameters between treated (*n* = 23) and treatment‐naïve (*n* = 18) patients. We did not detect clear variations in any of the CCM parameters (all *p* > 0.05), though these findings should be interpreted cautiously given the limited subgroup sizes (Table 5).

**TABLE 5:** Comparison of CCM parameters between treated and treatment‐naïve ATTRv‐PN patients.

	Treatment subgroup		*p*
	Treatment‐naïve (*n* = 18)	Treated (*n* = 23)	
CNFL (mm/mm^2^)	10.95 ± 5.59	8.73 ± 5.11	0.18
CNFD (no./mm^2^)	25.14 ± 9.94	22.40 ± 10.76	0.24
CNBD (no./mm^2^)	31.68 ± 24.21	21.01 ± 17.27	0.17
IWFL (mm/mm^2^)	11.86 ± 6.79	9.17 ± 4.32	0.20
IWFD (no./mm^2^)	23.44 ± 9.65	20.55 ± 7.44	0.18
IWBD (no./mm^2^)	50.95 ± 40.13	27.73 ± 21.60	0.11
CLCD (no./mm^2^)	26.45 ± 32.58	31.27 ± 23.26	0.17
IWLCD (no./mm^2^)	62.71 ± 75.60	36.02 ± 36.83	0.08

Abbreviations: CLCD, corneal Langerhans cell density; CNBD, corneal nerve branch density; CNFD, corneal nerve fiber density; CNFL, corneal nerve fiber length; IWBD, inferior whorl nerve branch density; IWFD, inferior whorl fiber density; IWFL, inferior whorl corneal nerve fiber length; IWLCD, inferior whorl Langerhans cell density.

### 3.3 CCM Parameters in Carriers

**FIGURE 2:**
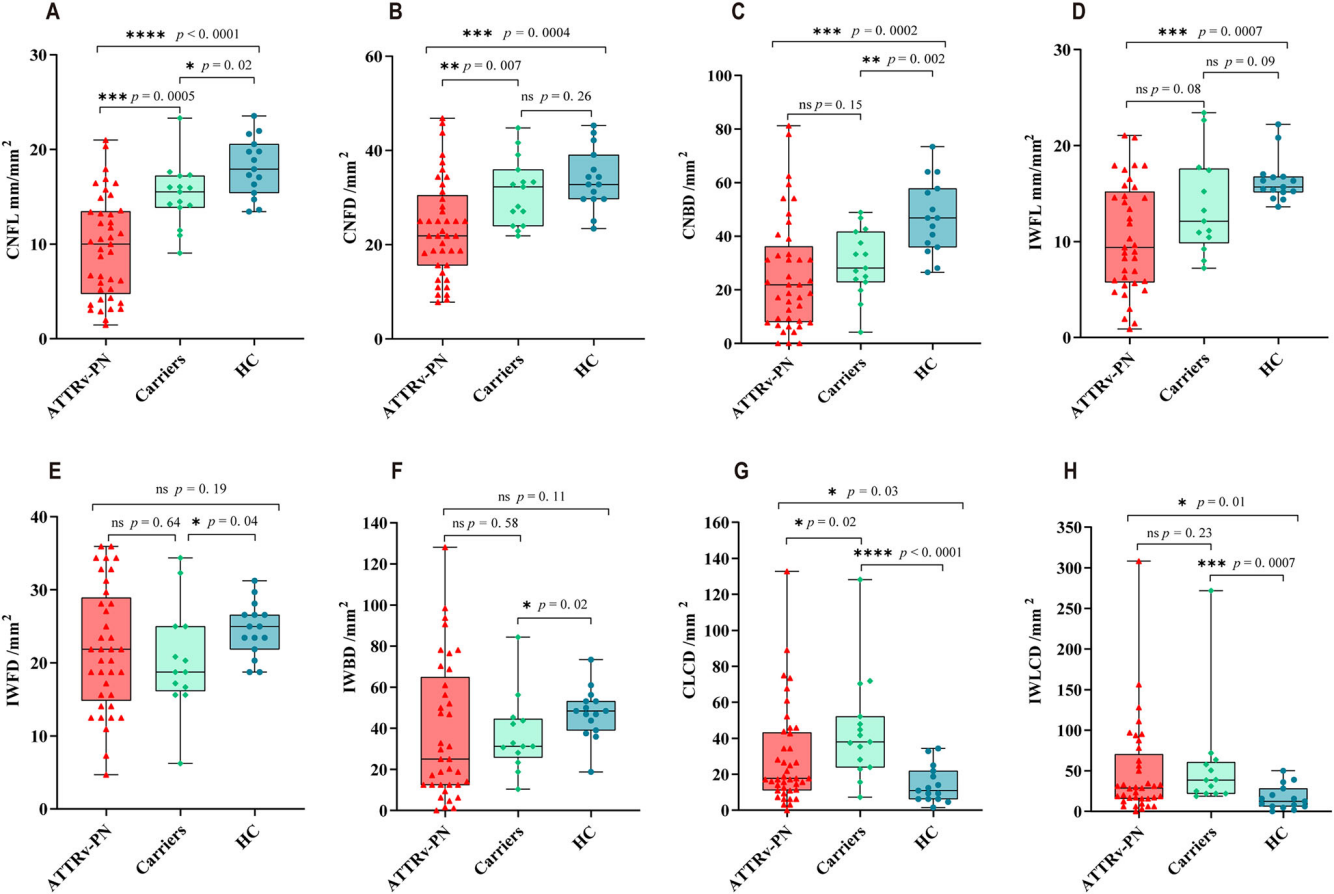
Comparison of CCM parameters between ATTRv‐PN patients, presymptomatic carriers, and healthy controls (HC). *p* values are indicated as follows: *ns*, not significant; * p < 0.05; **p < 0.01; *** *p* < 0.001; **** *p* < 0.0001.

The CCM evaluation showed CNFL (*p* = 0.02), CNBD (*p* = 0.002), IWFD (*p* = 0.04), and IWBD (*p* = 0.02) were lower in carriers than in the HC, while CNFD (*p* = 0.26) and IWFL (*p* = 0.09) were not (Figure  2A–F). The CLCD (*p* < 0.0001) and IWLCD (*p* = 0.0007) were significantly higher in carriers than in the HC group (Figure 2G,H). The CNFL (*p* = 0.0005) and CNFD (*p* = 0.007) were significantly lower in patients than in carriers (Figure 2A,B). The CLCD levels (*p* = 0.02) were higher in carriers than in patients (Figure 2G). As for other parameters, no statistically significant differences were detected (all *p* > 0.05).

### 3.4 Relationships to the Severity of Neuropathy and Autonomic Symptoms

**FIGURE 3:**
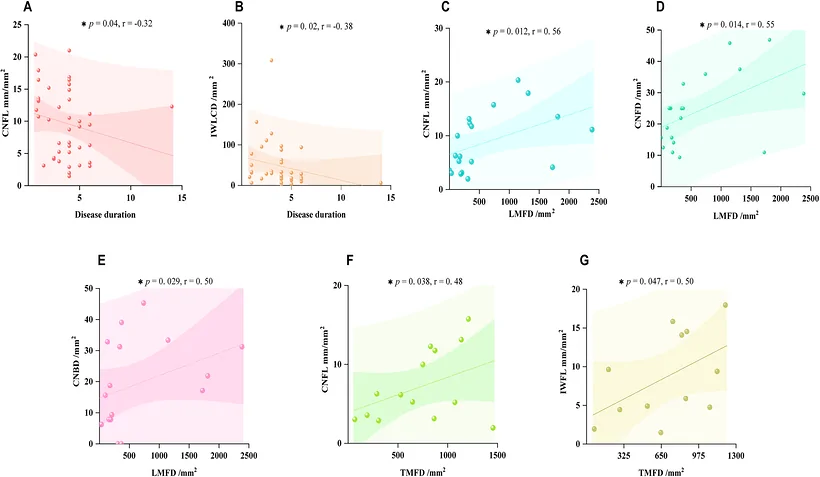
Correlations of CCM parameters with clinical and nerve fiber measures. (A, B) CNFL and IWLCD correlated with disease duration. (C–E) CNFD, CNBD, and CNFL correlated with a large myelinated fiber density (LMFD). (F, G) CNFL and IWFL correlated with a total myelinated fiber density (TMFD).

The NIS‐LL score (58.86 ± 37.00) and the COMPASS‐31 score (39.93 ± 22.86) of the patients were both elevated. CNFL (*r* = −0.32, *p* = 0.04) and IWLCD (*r* = −0.38, *p* = 0.02) were correlated with disease duration (Figure 3A,B). The analysis revealed no significant correlational relationship between CCM parameters and either the NIS‐LL score or the COMPASS‐31 score (all *p* > 0.05) in ATTRv‐PN patients.

### 3.5 The Nerve Conduction Study

The motor nerve conduction velocity (MCV) of the median nerve and ulnar nerve was 47.06 ± 10.21 and 47.91 ± 10.50 m/s, respectively. The CMAP amplitude of the median nerve and ulnar nerve was 7.28 ± 9.53 and 6.47 ± 4.21 mV, respectively. Severe sensory nerve injury occurred in 31/41 patients, and most of the sensory nerve action potentials (SNAPs) were not elicited. The SNAP amplitude of other patients was significantly decreased. There was a positive correlation between IWBD and CMAP amplitude of the ulnar nerve (*r* = 0.428, *p* = 0.020). The analysis revealed no significant correlations between CCM parameters and other electrophysiological data (all *p* > 0.05).

### 3.6 Sural Nerve Pathology Study

The mean of small myelinated fiber density (SMFD), large myelinated fiber density (LMFD), and total myelinated fiber density (TMFD) was 1011.66 ± 985.38, 622.64 ± 704.01, and 1634.30 ± 1669.78 no./mm^2^. The myelinated nerve fiber density of all patients showed a substantial decrease.

CNFL (*r* = 0.56, *p* = 0.012), CNFD (*r* = 0.55, *p* = 0.014), and CNBD (*r* = 0.50, *p* = 0.029) were positively correlated with LMFD (Figure 3 C–E), so were CNFL (*r* = 0.48, *p* = 0.038) and IWFL (*r* = 0.50, *p* = 0.047) with TMFD (Figure 3 F,G). SMFD was not correlated with the CCM parameters (*p* > 0.05).

## 4 Discussion

Transmitted in an autosomal dominant pattern, ATTRv‐PN is a rare peripheral neuropathy involving the peripheral nervous system, with potential additional involvement of the heart and other organs (Adams, Ando et al. 2021). Mutations in the *TTR* gene lead to the production of misfolded transthyretin proteins, which aggregate into amyloid fibrils and subsequently deposit in multiple organs and tissues. This condition is progressive and life‐threatening and can manifest as either small fiber neuropathy or a sensorimotor axonal neuropathy (Jiao et al. 2025; Planté‐Bordeneuve and Said 2011). Its rarity and diagnostic complexity often lead to misdiagnosis with other neuropathies, such as chronic inflammatory demyelinating polyneuropathy (CIDP) or diabetic peripheral neuropathy (DPN) (Du et al. 2022). Disease‐modifying treatments (DMTs) such as gene silencer and TTR stabilizer are effective (Plante‐Bordeneuve 2018). Initiating treatment in the early stages of the disease can delay or halt disease progression and may alter the clinical course. Therefore, early recognition and diagnosis of this disorder are crucial (Adams, Ando et al. 2021).

As a non‐invasive ocular imaging modality, CCM serves as a rapid, repeatable, and cost‐effective biomarker for imaging corneal nerve fibers (Petropoulos et al. 2020). CCM allows for the identification of small nerve fiber damage (Frizziero et al. 2023). Compared with previous studies (Thimm et al. 2023; Zhang et al. 2021; Alcantara et al. 2024), ours was the largest ATTRv‐PN cohort with CCM examinations, including 21 different *TTR* genotypes. We confirmed reduced CNFL, CNFD, CNBD, IWFL, IWFD, and IWBD in patients with ATTRv‐PN, indicating the comprehensive damage of corneal small nerve fibers caused by the disease. In fact, in other forms of the disease, such as hereditary transthyretin amyloidosis with cardiomyopathy (ATTRv‐CM), wild‐type transthyretin amyloidosis (ATTRwt), the morphological abnormality of corneal small nerve fibers can also be detected (Thimm et al. 2023; Frizziero et al. 2023), suggesting the high sensitivity of CCM for evaluating the small fiber neuropathy in transthyretin amyloidosis. We are not sure whether CCM can be used for differential diagnosis between ATTRv‐PN and mimics, as CIDP and DPN can also exhibit CCM abnormalities (Fleischer et al. 2021; Ferdousi et al. 2020; Klimas et al. 2024). Another study from China, containing two *TTR* A97S families, proved more severe damage to corneal small nerve fibers in cases in Coutinho stage II than those in Coutinho stage I (Zhang et al. 2021). Surprisingly, in our study, none of the CCM parameters was statistically different between cases in Stage I and those in Stage II. Despite the influence of more diverse genotypes and fewer cases from the same family in our cohort, this result could possibly indicate the potential severe damage to corneal small nerve fibers in the early stage of ATTRv‐PN, suggesting the importance of early diagnosis and disease‐modifying therapy. There was no statistical difference in CCM parameters between early‐onset cases and late‐onset cases, though in the two groups, different clinical and pathological features were identified (Koike et al. 2009; Koike et al. 2002).

We analyzed the potential impact of DMTs on CCM parameters and found no distinct variations based on treatment status, which was left uninvestigated by previous studies. In our view, this absence of statistical significance should not be interpreted as an absence of therapeutic efficacy for DMTs. The strictly cross‐sectional design of our study inherently precludes the evaluation of longitudinal treatment effects on small nerve fibers. Although treatment status did not demonstrate a marked effect in our current baseline comparisons, prospective, longitudinal studies with larger, uniformly treated cohorts are needed to elucidate the precise impact of DMTs on CCM parameters.

We confirmed the abnormalities of most of the CCM parameters except CNFD and IWFL for presymptomatic carriers. Previous studies have shown that presymptomatic carriers undergo changes in sural nerve pathology and MR neurography compared with healthy controls (Fernandes et al. 2019; Kollmer et al. 2017), and our previous study proved the reduced serum TTR tetramer concentration in *TTR* E61K presymptomatic carriers (Chu et al. 2022), indicating the early abnormal changes in these carriers. The timing of treatment for presymptomatic carriers is critical but complicated, but studies on early treatment for presymptomatic carriers are lacking. While our study has revealed early abnormalities in corneal nerves among carriers, these findings alone are insufficient to guide therapeutic decision‐making.

We found increases in the CLCD and IWLCD in ATTRv‐PN patients and carriers, with CLCD differing significantly between the three groups, and a negative correlation between IWLCD and disease duration. CLCD was reportedly elevated around the central whorl among early‐phase patients, but it was reduced to some extent in progressive patients (Zhang et al. 2021). Langerhans cells can show a significant increase during the asymptomatic phase, seemingly decreasing gradually as the disease progresses. While CCM alone cannot establish direct causality, this increase may reflect immune activation associated with disease processes. This finding aligns with recent research that mutant transthyretin disrupts the blood‐nerve barrier and triggers macrophage‐mediated neuroinflammation (Chao et al. 2025). More investigation into the specific mechanisms of inflammation is warranted to elucidate the potential of anti‐inflammatory therapy as a novel strategy for ATTRv‐PN.

We confirmed the relationship between CNFL and the disease duration, which was also reported in other studies (Plante‐Bordeneuve 2018; Planté‐Bordeneuve and Said 2011). We found no correlation between CCM parameters and the NIS‐LL score or the COMPASS‐31 score in ATTRv‐PN patients. In other neuropathies such as CIDP and DPN, previous studies have demonstrated that CCM is linked to disease severity (Kalteniece et al. 2022; Stettner et al. 2016). Considering the robust positive correlations observed between CCM parameters and myelinated nerve fiber density in sural nerve biopsies in our cohort, the lack of correlation with the NIS‐LL and COMPASS‐31 may reflect the inherent limitations of these clinical scores rather than a deficiency in CCM. Our study found that CCM parameters were not correlated with SMFD in the sural nerve. Several studies have shown that ATTRv‐PN patients with lower intraepidermal nerve fiber density (IENFD) tend to have reduced CNFL, but there is no evidence to suggest a linear correlation between them (Petropoulos et al. 2021; Gylfadottir et al. 2023). Similarly, in patients with DPN, IENFD is not correlated with CCM parameters. Interestingly, we found CNFD, CNBD, CNFL were correlated with LMFD, while CNFL and IWFL were correlated with TMFD, indicating some synchronicity between the damage to corneal nerve fibers and the decrease of sural nerve large myelinated fibers, though the related mechanism was still unknown.

A limitation of this study was the relatively small sample size, and adjusted analyses lacked sufficient statistical power and were not performed. Future larger studies are needed to isolate the disease‐specific impacts on corneal nerves. Furthermore, our exploratory subgroup analyses were limited by small sample sizes and genetic heterogeneity. Importantly, while we justified not applying strict multiple comparison corrections to avoid inflating Type II errors, this approach inherently increases the risk of false‐positive findings. Therefore, these exploratory results must be interpreted with caution, and larger multicenter cohorts are required to adequately power these comparisons.

## 5 Conclusion

As an in vivo, noninvasive imaging technique, CCM facilitates the detailed evaluation of corneal nerve fiber morphology in both symptomatic ATTRv‐PN individuals and asymptomatic mutation carriers. Our study has substantiated that CCM parameters of corneal nerve fibers in ATTRv‐PN patients and presymptomatic carriers show significant abnormalities.

## Author Contributions

**Yize Li** conducted statistical analyses and prepared the manuscript. **Wenjing Song** performed CCM. **Xujun Chu**, **Ping Li**, **Zhenyu** **Li**, **He Lv**, **Wei Zhang**, **Zhaoxia Wang**, **Yun Yuan**, and **Lingchao Meng** completed the diagnosis of ATTRv‐PN. **Kang Du** and **Difang Shi** collected and analyzed the data. **Lingchao Meng** and **Yuan Wu**, conceived the study and critically revised the manuscript. All authors read and approved the final manuscript.

## Funding

This study was supported by the National High Level Hospital Clinical Research Funding (Interdepartmental Research Project of Peking University First Hospital) (2024IR12).

## Ethics

The studies involving human participants followed ethical principles in accordance with the Helsinki Declaration and were approved by the Ethic Committee of Peking University First Hospital (2024‐095‐002).

## Consent

The written informed consent for publication of identifying images or other personal or clinical details was obtained from the participants in this study.

## Conflicts of Interest

The authors declare no conflicts of interest.

## Data Availability

The original contributions presented in the study are included in the article/supplementary material, and further inquiries can be directed to the corresponding authors.
